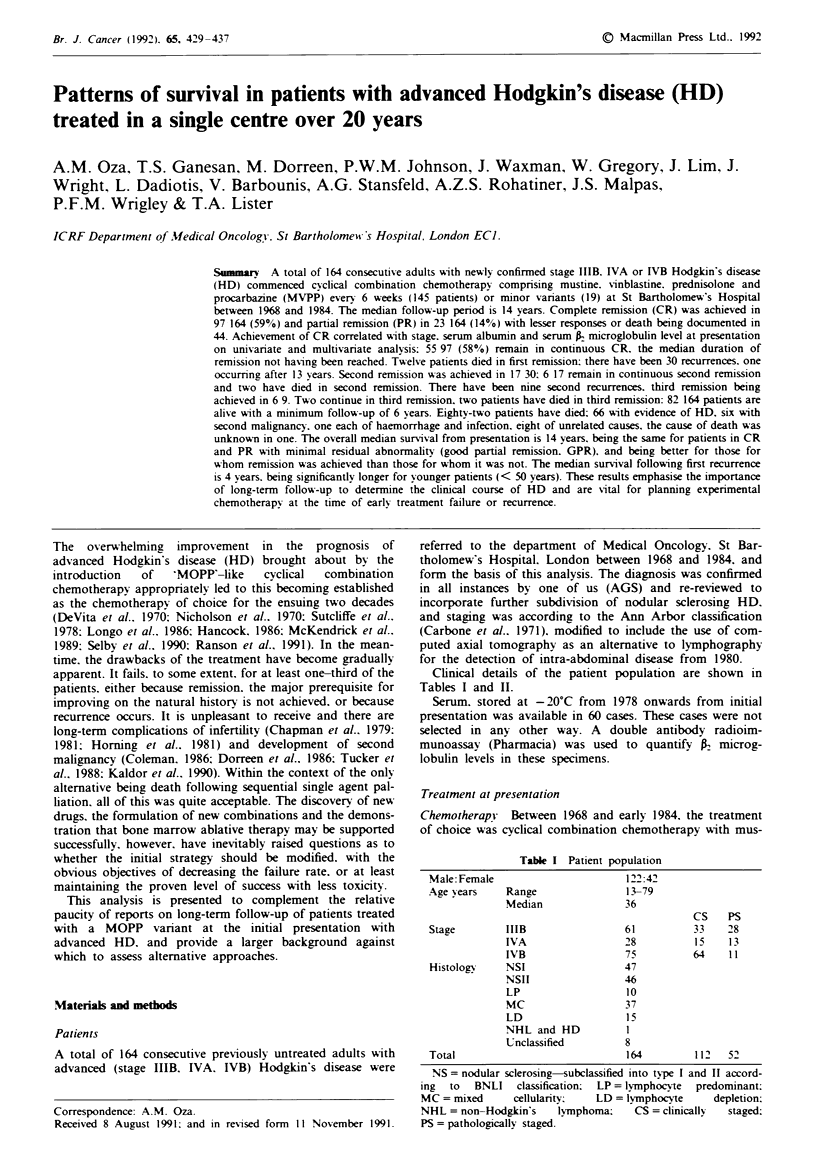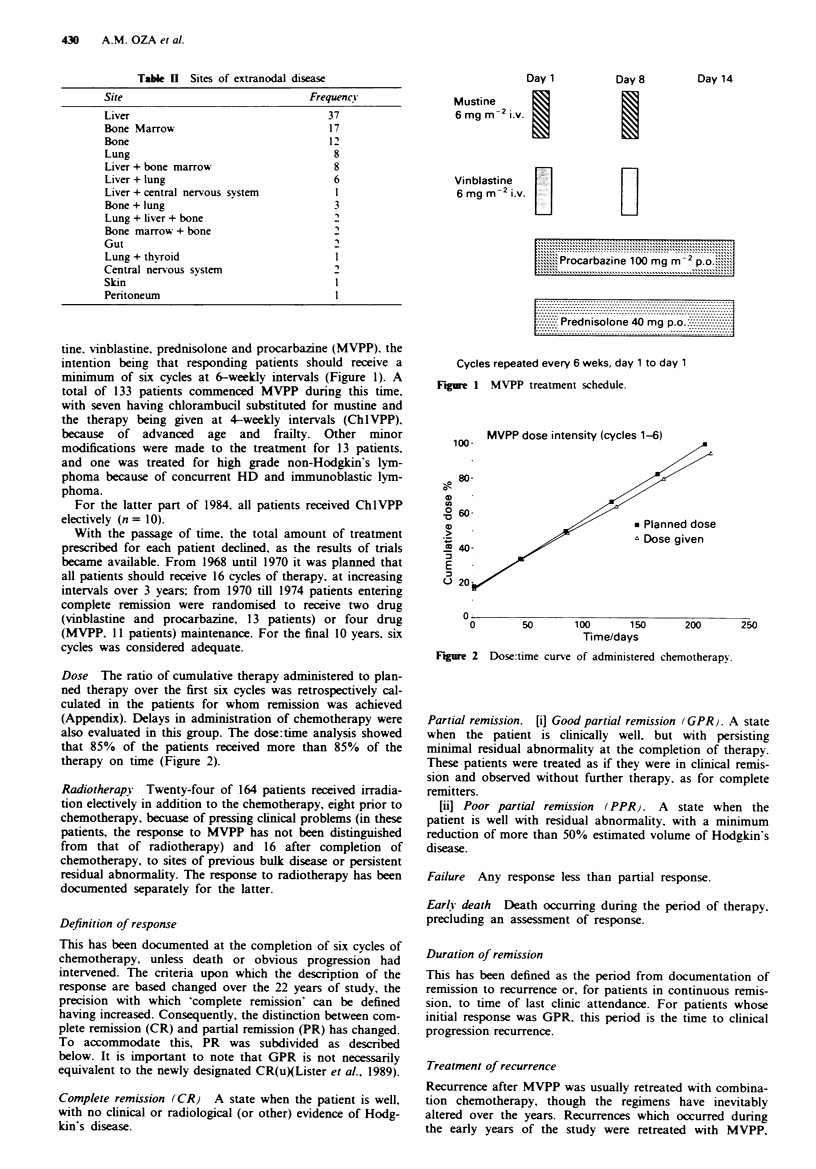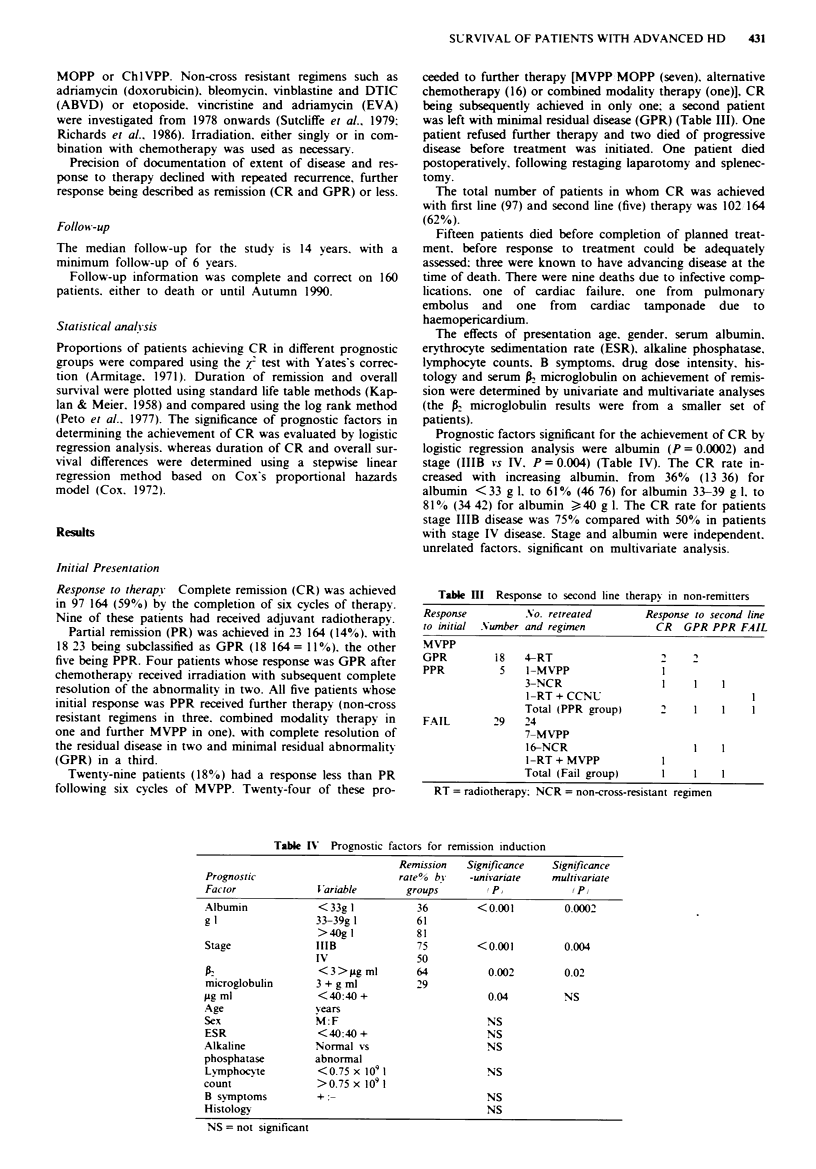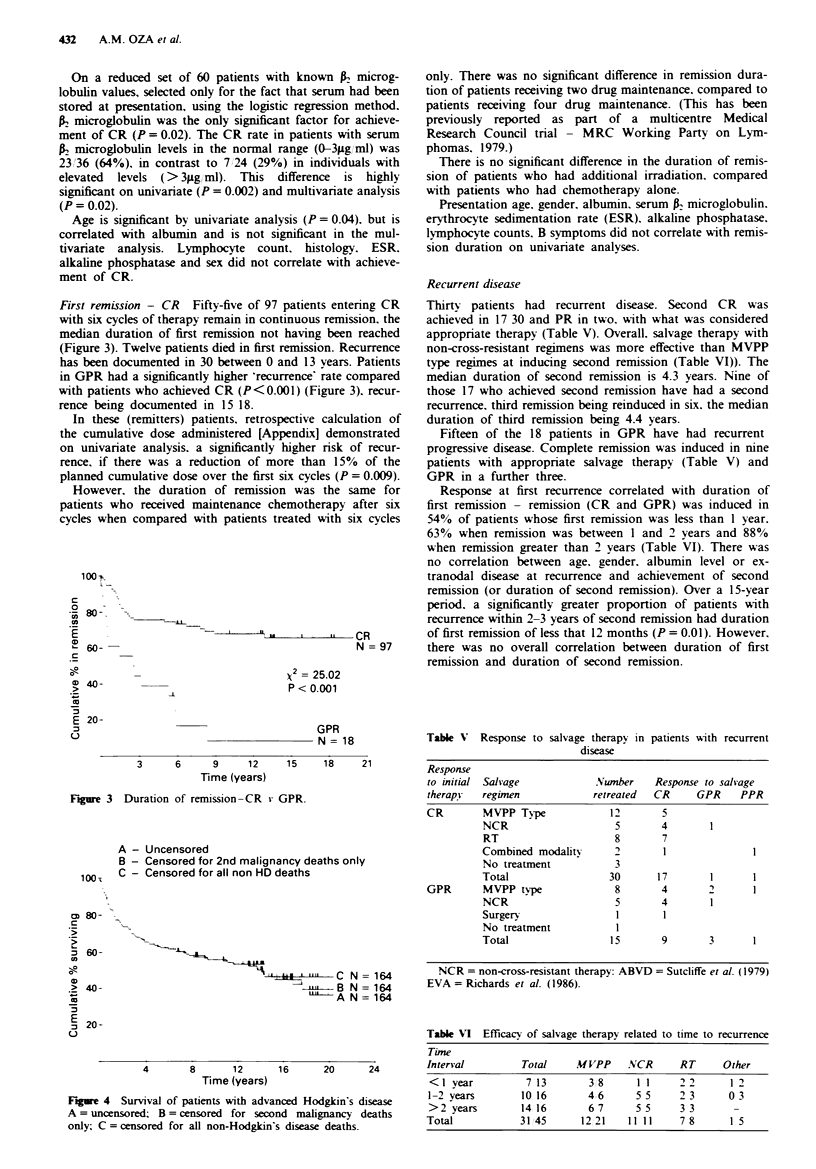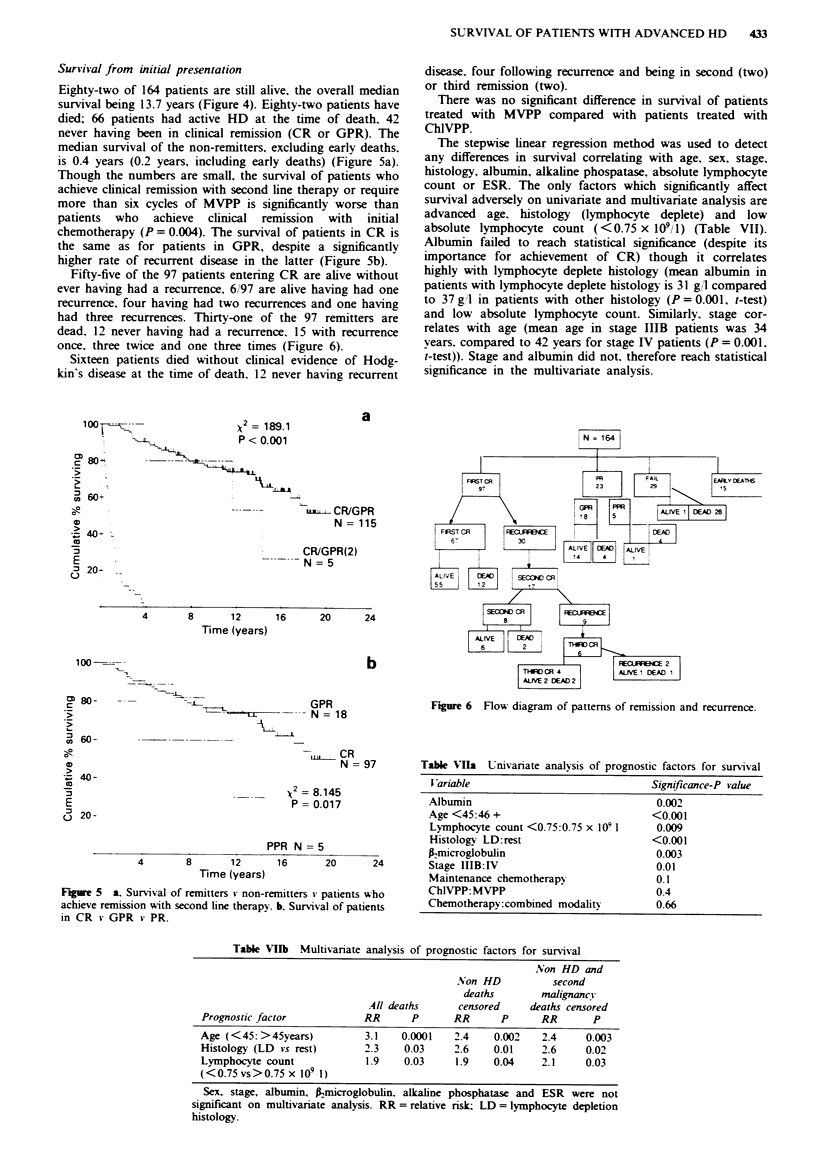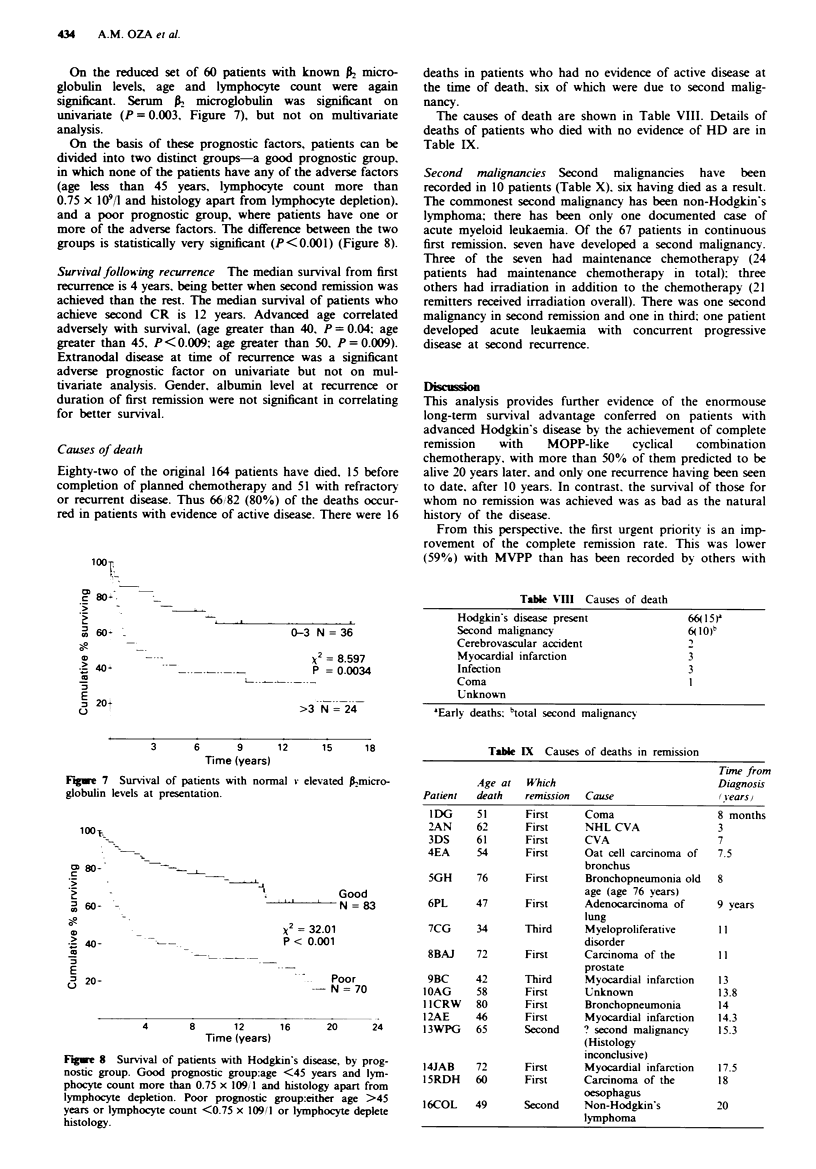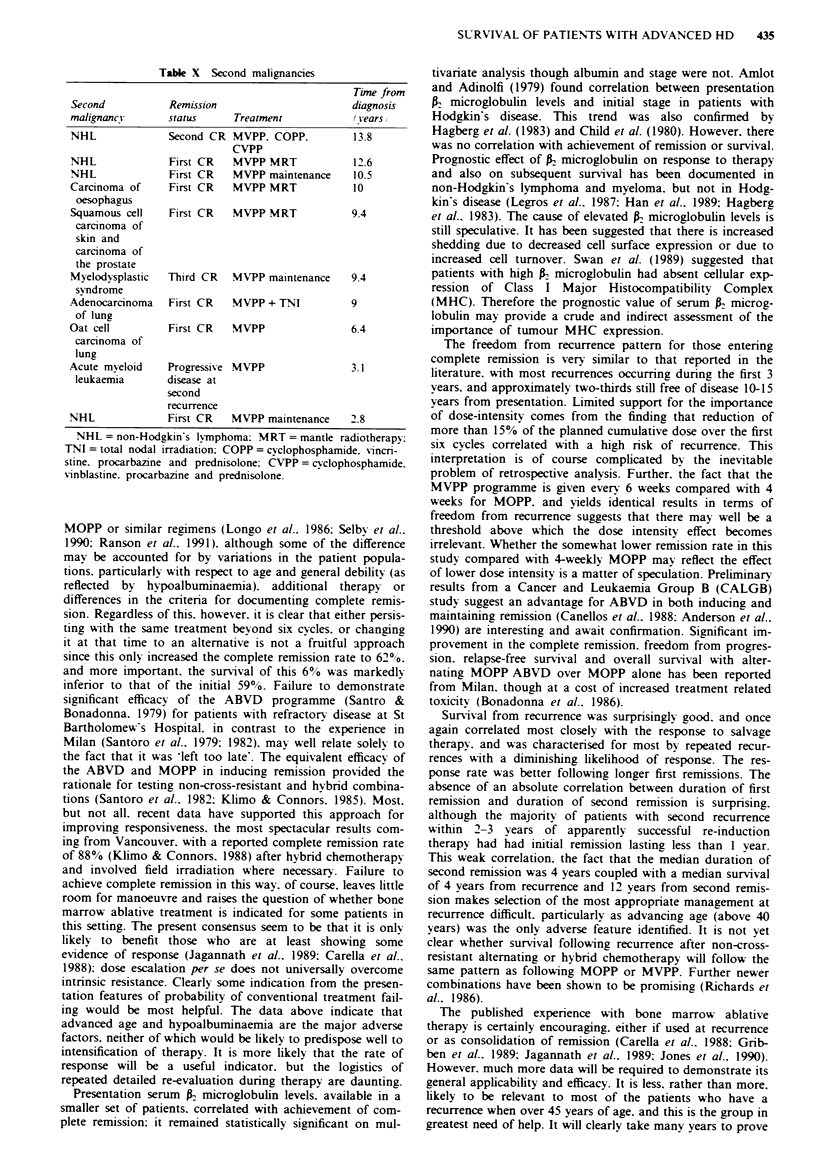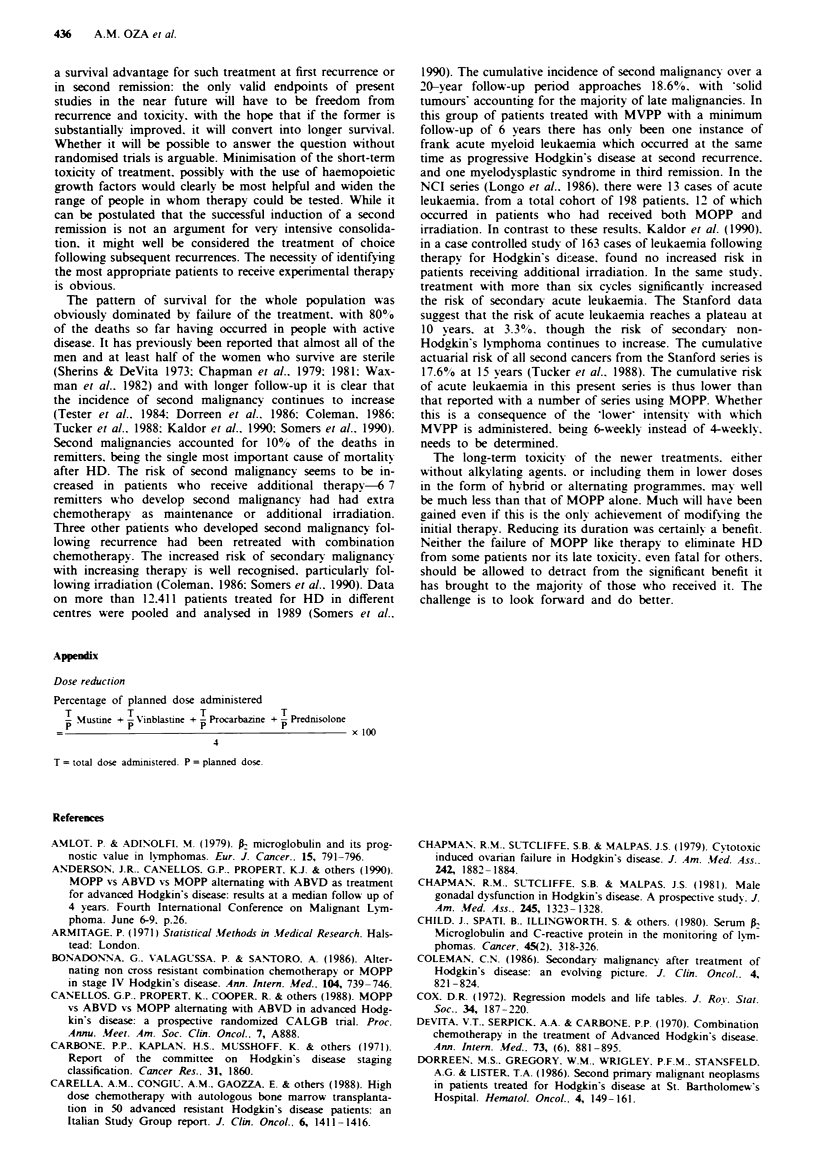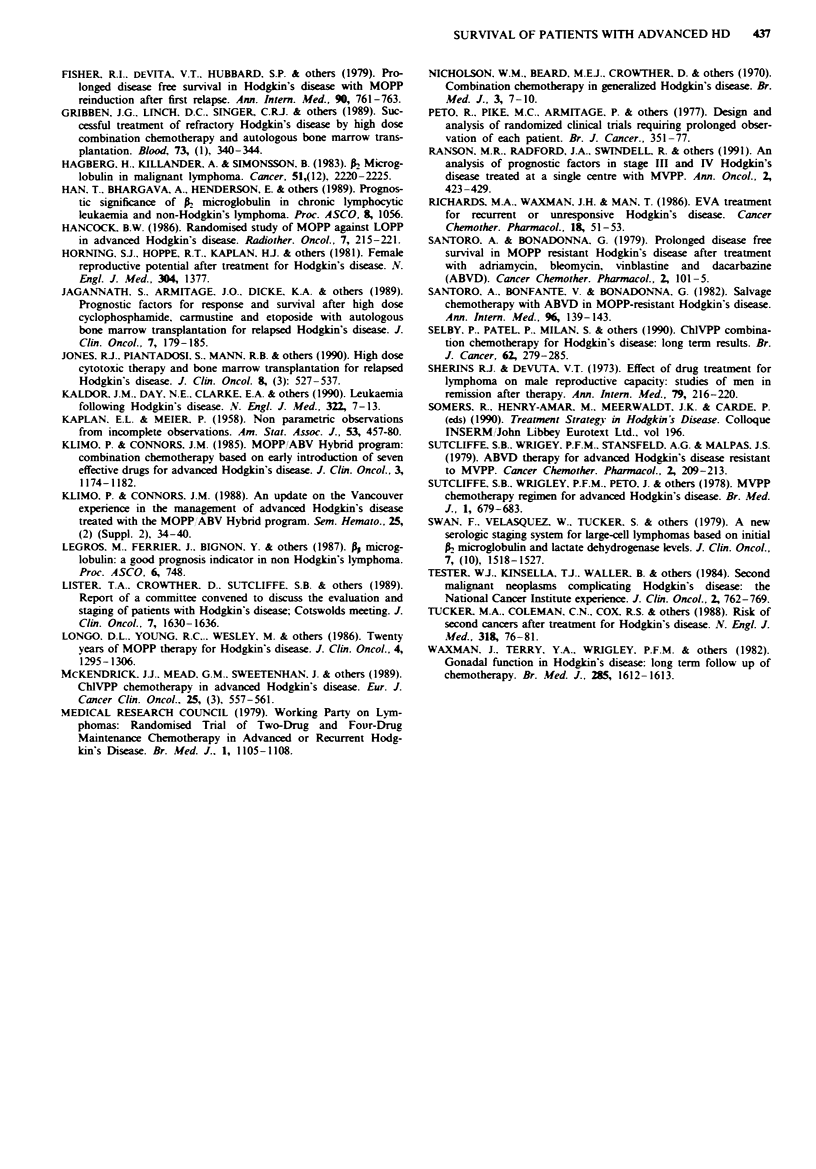# Patterns of survival in patients with advanced Hodgkin's disease (HD) treated in a single centre over 20 years.

**DOI:** 10.1038/bjc.1992.88

**Published:** 1992-03

**Authors:** A. M. Oza, T. S. Ganesan, M. Dorreen, P. W. Johnson, J. Waxman, W. Gregory, J. Lim, J. Wright, L. Dadiotis, V. Barbounis

**Affiliations:** ICRF Department of Medical Oncology, St Bartholomew's Hospital, London.

## Abstract

A total of 164 consecutive adults with newly confirmed stage IIIB, IVA or IVB Hodgkin's disease (HD) commenced cyclical combination chemotherapy comprising mustine, vinblastine, prednisolone and procarbazine (MVPP) every 6 weeks (145 patients) or minor variants (19) at St Bartholomew's Hospital between 1968 and 1984. The median follow-up period is 14 years. Complete remission (CR) was achieved in 97/164 (59%) and partial remission (PR) in 23/164 (14%) with lesser responses or death being documented in 44. Achievement of CR correlated with stage, serum albumin and serum beta2 microglobulin level at presentation on univariate and multivariate analysis; 55/97 (58%) remain in continuous CR, the median duration of remission not having been reached. Twelve patients died in first remission; there have been 30 recurrences, one occurring after 13 years. Second remission was achieved in 17/30; 6/17 remain in continuous second remission and two have died in second remission. There have been nine second recurrences, third remission being achieved in 6/9. Two continue in third remission, two patients have died in third remission: 82/164 patients are alive with a minimum follow-up of 6 years. Eighty-two patients have died; 66 with evidence of HD, six with second malignancy, one each of haemorrhage and infection, eight of unrelated causes, the cause of death was unknown in one. The overall median survival from presentation is 14 years, being the same for patients in CR and PR with minimal residual abnormality (good partial remission, GPR), and being better for those for whom remission was achieved than those for whom it was not. The median survival following first recurrence is 4 years, being significantly longer for younger patients (less than 50 years). These results emphasise the importance of long-term follow-up to determine the clinical course of HD and are vital for planning experimental chemotherapy at the time of early treatment failure or recurrence.


Br. J. Cancer (1992), 65, 429-437 © Macmillan Press Ltd., 1992

### Patterns of survival in patients with advanced Hodgkin's disease (HD) treated in a single centre over 20 years

A.M. Oza, T.S. Ganesan, M. Dorreen, P.W.M. Johnson, J. Waxman, W. Gregory, J. Lim, J. Wright, L. Dadiotis, V. Barbounis, A.G. Stansfeld, A.Z.S. Rohatiner, J.S. Malpas, P.F.M. Wrigley & T.A. Lister

*ICRF Department of Medical Oncology, St Bartholomew's Hospital, London EC1.*

**Summary** A total of 164 consecutive adults with newly confirmed stage IIIB, IVA or IVB Hodgkin's disease (HD) commenced cyclical combination chemotherapy comprising mustine, vinblastine, prednisolone and procarbazine (MVPP) every 6 weeks (145 patients) or minor variants (19) at St Bartholomew's Hospital between 1968 and 1984. The median follow-up period is 14 years. Complete remission (CR) was achieved in 97 164 (59%) and partial remission (PR) in 23 164 (14%) with lesser responses or death being documented in 44. Achievement of CR correlated with stage, serum albumin and serum $\beta_2$ microglobulin level at presentation on univariate and multivariate analysis; 55 97 (58%) remain in continuous CR, the median duration of remission not having been reached. Twelve patients died in first remission; there have been 30 recurrences, one occurring after 13 years. Second remission was achieved in 17 30; 6 17 remain in continuous second remission and two have died in second remission. There have been nine second recurrences, third remission being achieved in 6 9. Two continue in third remission, two patients have died in third remission: 82 164 patients are alive with a minimum follow-up of 6 years. Eighty-two patients have died; 66 with evidence of HD, six with second malignancy, one each of haemorrhage and infection, eight of unrelated causes, the cause of death was unknown in one. The overall median survival from presentation is 14 years, being the same for patients in CR and PR with minimal residual abnormality (good partial remission, GPR), and being better for those for whom remission was achieved than those for whom it was not. The median survival following first recurrence is 4 years, being significantly longer for younger patients (< 50 years). These results emphasise the importance of long-term follow-up to determine the clinical course of HD and are vital for planning experimental chemotherapy at the time of early treatment failure or recurrence.

The overwhelming improvement in the prognosis of advanced Hodgkin's disease (HD) brought about by the introduction of 'MOPP'-like cyclical combination chemotherapy appropriately led to this becoming established as the chemotherapy of choice for the ensuing two decades (DeVita *et al.*, 1970; Nicholson *et al.*, 1970; Sutcliffe *et al.*, 1978; Longo *et al.*, 1986; Hancock, 1986; McKendrick *et al.*, 1989; Selby *et al.*, 1990; Ranson *et al.*, 1991). In the meantime, the drawbacks of the treatment have become gradually apparent. It fails, to some extent, for at least one-third of the patients, either because remission, the major prerequisite for improving on the natural history is not achieved, or because recurrence occurs. It is unpleasant to receive and there are long-term complications of infertility (Chapman *et al.*, 1979; 1981; Horning *et al.*, 1981) and development of second malignancy (Coleman, 1986; Dorreen *et al.*, 1986; Tucker *et al.*, 1988; Kaldor *et al.*, 1990). Within the context of the only alternative being death following sequential single agent palliation, all of this was quite acceptable. The discovery of new drugs, the formulation of new combinations and the demonstration that bone marrow ablative therapy may be supported successfully, however, have inevitably raised questions as to whether the initial strategy should be modified, with the obvious objectives of decreasing the failure rate, or at least maintaining the proven level of success with less toxicity.

This analysis is presented to complement the relative paucity of reports on long-term follow-up of patients treated with a MOPP variant at the initial presentation with advanced HD, and provide a larger background against which to assess alternative approaches.

### Materials and methods

### Patients

A total of 164 consecutive previously untreated adults with advanced (stage IIIB, IVA, IVB) Hodgkin's disease were referred to the department of Medical Oncology, St Bartholomew's Hospital, London between 1968 and 1984, and form the basis of this analysis. The diagnosis was confirmed in all instances by one of us (AGS) and re-reviewed to incorporate further subdivision of nodular sclerosing HD, and staging was according to the Ann Arbor classification (Carbone *et al.*, 1971), modified to include the use of computed axial tomography as an alternative to lymphography for the detection of intra-abdominal disease from 1980.

Clinical details of the patient population are shown in Tables I and II.

Serum, stored at −20°C from 1978 onwards from initial presentation was available in 60 cases. These cases were not selected in any other way. A double antibody radioimmunoassay (Pharmacia) was used to quantify $\beta_2$ microglobulin levels in these specimens.

### Treatment at presentation

*Chemotherapy* Between 1968 and early 1984, the treatment of choice was cyclical combination chemotherapy with mus-

**Table I** Patient population

| | | | | |
|---|---|---|---|---|
| Male:Female | | 122:42 | | |
| Age years | Range | 13–79 | | |
| | Median | 36 | | |
| | | | CS | PS |
| Stage | IIIB | 61 | 33 | 28 |
| | IVA | 28 | 15 | 13 |
| | IVB | 75 | 64 | 11 |
| Histology | NSI | 47 | | |
| | NSII | 46 | | |
| | LP | 10 | | |
| | MC | 37 | | |
| | LD | 15 | | |
| | NHL and HD | 1 | | |
| | Unclassified | 8 | | |
| Total | | 164 | 112 | 52 |

NS = nodular sclerosing—subclassified into type I and II according to BNLI classification; LP = lymphocyte predominant; MC = mixed cellularity; LD = lymphocyte depletion; NHL = non-Hodgkin's lymphoma; CS = clinically staged; PS = pathologically staged.

Correspondence: A.M. Oza.
Received 8 August 1991; and in revised form 11 November 1991.

**Table II** Sites of extranodal disease

| *Site* | *Frequency* |
|---|---|
| Liver | 37 |
| Bone Marrow | 17 |
| Bone | 12 |
| Lung | 8 |
| Liver + bone marrow | 8 |
| Liver + lung | 6 |
| Liver + central nervous system | 1 |
| Bone + lung | 3 |
| Lung + liver + bone | 2 |
| Bone marrow + bone | 2 |
| Gut | 2 |
| Lung + thyroid | 1 |
| Central nervous system | 2 |
| Skin | 1 |
| Peritoneum | 1 |

tine, vinblastine, prednisolone and procarbazine (MVPP), the intention being that responding patients should receive a minimum of six cycles at 6-weekly intervals (Figure 1). A total of 133 patients commenced MVPP during this time, with seven having chlorambucil substituted for mustine and the therapy being given at 4-weekly intervals (ChlVPP), because of advanced age and frailty. Other minor modifications were made to the treatment for 13 patients, and one was treated for high grade non-Hodgkin's lymphoma because of concurrent HD and immunoblastic lymphoma.

For the latter part of 1984, all patients received ChlVPP electively ($n = 10$).

With the passage of time, the total amount of treatment prescribed for each patient declined, as the results of trials became available. From 1968 until 1970 it was planned that all patients should receive 16 cycles of therapy, at increasing intervals over 3 years; from 1970 till 1974 patients entering complete remission were randomised to receive two drug (vinblastine and procarbazine, 13 patients) or four drug (MVPP, 11 patients) maintenance. For the final 10 years, six cycles was considered adequate.

*Dose* The ratio of cumulative therapy administered to planned therapy over the first six cycles was retrospectively calculated in the patients for whom remission was achieved (Appendix). Delays in administration of chemotherapy were also evaluated in this group. The dose:time analysis showed that 85% of the patients received more than 85% of the therapy on time (Figure 2).

*Radiotherapy* Twenty-four of 164 patients received irradiation electively in addition to the chemotherapy, eight prior to chemotherapy, becuase of pressing clinical problems (in these patients, the response to MVPP has not been distinguished from that of radiotherapy) and 16 after completion of chemotherapy, to sites of previous bulk disease or persistent residual abnormality. The response to radiotherapy has been documented separately for the latter.

### *Definition of response*

This has been documented at the completion of six cycles of chemotherapy, unless death or obvious progression had intervened. The criteria upon which the description of the response are based changed over the 22 years of study, the precision with which 'complete remission' can be defined having increased. Consequently, the distinction between complete remission (CR) and partial remission (PR) has changed. To accommodate this, PR was subdivided as described below. It is important to note that GPR is not necessarily equivalent to the newly designated CR(u)(Lister *et al.*, 1989).

*Complete remission (CR)* A state when the patient is well, with no clinical or radiological (or other) evidence of Hodgkin's disease.

**Figure 1** MVPP treatment schedule.

**Figure 2** Dose:time curve of administered chemotherapy.

*Partial remission.* [i] *Good partial remission (GPR).* A state when the patient is clinically well, but with persisting minimal residual abnormality at the completion of therapy. These patients were treated as if they were in clinical remission and observed without further therapy, as for complete remitters.

[ii] *Poor partial remission (PPR).* A state when the patient is well with residual abnormality, with a minimum reduction of more than 50% estimated volume of Hodgkin's disease.

*Failure* Any response less than partial response.

*Early death* Death occurring during the period of therapy, precluding an assessment of response.

### *Duration of remission*

This has been defined as the period from documentation of remission to recurrence or, for patients in continuous remission, to time of last clinic attendance. For patients whose initial response was GPR, this period is the time to clinical progression/recurrence.

### *Treatment of recurrence*

Recurrence after MVPP was usually retreated with combination chemotherapy, though the regimens have inevitably altered over the years. Recurrences which occurred during the early years of the study were retreated with MVPP,

MOPP or ChlVPP. Non-cross resistant regimens such as adriamycin (doxorubicin), bleomycin, vinblastine and DTIC (ABVD) or etoposide, vincristine and adriamycin (EVA) were investigated from 1978 onwards (Sutcliffe *et al.*, 1979; Richards *et al.*, 1986). Irradiation, either singly or in combination with chemotherapy was used as necessary.

Precision of documentation of extent of disease and response to therapy declined with repeated recurrence, further response being described as remission (CR and GPR) or less.

*Follow-up*

The median follow-up for the study is 14 years, with a minimum follow-up of 6 years.

Follow-up information was complete and correct on 160 patients, either to death or until Autumn 1990.

*Statistical analysis*

Proportions of patients achieving CR in different prognostic groups were compared using the $\chi^2$ test with Yates's correction (Armitage, 1971). Duration of remission and overall survival were plotted using standard life table methods (Kaplan & Meier, 1958) and compared using the log rank method (Peto *et al.*, 1977). The significance of prognostic factors in determining the achievement of CR was evaluated by logistic regression analysis, whereas duration of CR and overall survival differences were determined using a stepwise linear regression method based on Cox's proportional hazards model (Cox, 1972).

### Results

*Initial Presentation*

*Response to therapy* Complete remission (CR) was achieved in 97/164 (59%) by the completion of six cycles of therapy. Nine of these patients had received adjuvant radiotherapy.

Partial remission (PR) was achieved in 23/164 (14%), with 18/23 being subclassified as GPR (18/164 = 11%), the other five being PPR. Four patients whose response was GPR after chemotherapy received irradiation with subsequent complete resolution of the abnormality in two. All five patients whose initial response was PPR received further therapy (non-cross resistant regimens in three, combined modality therapy in one and further MVPP in one), with complete resolution of the residual disease in two and minimal residual abnormality (GPR) in a third.

Twenty-nine patients (18%) had a response less than PR following six cycles of MVPP. Twenty-four of these proceeded to further therapy [MVPP/MOPP (seven), alternative chemotherapy (16) or combined modality therapy (one)], CR being subsequently achieved in only one; a second patient was left with minimal residual disease (GPR) (Table III). One patient refused further therapy and two died of progressive disease before treatment was initiated. One patient died postoperatively, following restaging laparotomy and splenectomy.

The total number of patients in whom CR was achieved with first line (97) and second line (five) therapy was 102/164 (62%).

Fifteen patients died before completion of planned treatment, before response to treatment could be adequately assessed; three were known to have advancing disease at the time of death. There were nine deaths due to infective complications, one of cardiac failure, one from pulmonary embolus and one from cardiac tamponade due to haemopericardium.

The effects of presentation age, gender, serum albumin, erythrocyte sedimentation rate (ESR), alkaline phosphatase, lymphocyte counts, B symptoms, drug dose intensity, histology and serum $\beta_2$ microglobulin on achievement of remission were determined by univariate and multivariate analyses (the $\beta_2$ microglobulin results were from a smaller set of patients).

Prognostic factors significant for the achievement of CR by logistic regression analysis were albumin ($P = 0.0002$) and stage (IIIB *vs* IV, $P = 0.004$) (Table IV). The CR rate increased with increasing albumin, from 36% (13/36) for albumin <33 g/l, to 61% (46/76) for albumin 33–39 g/l, to 81% (34/42) for albumin ≥40 g/l. The CR rate for patients stage IIIB disease was 75% compared with 50% in patients with stage IV disease. Stage and albumin were independent, unrelated factors, significant on multivariate analysis.

**Table III** Response to second line therapy in non-remitters

| *Response to initial* | *Number* | *No. retreated and regimen* | *Response to second line CR* | *GPR* | *PPR* | *FAIL* |
|---|---|---|---|---|---|---|
| MVPP | | | | | | |
| GPR | 18 | 4–RT | 2 | 2 | | |
| PPR | 5 | 1–MVPP | 1 | | | |
| | | 3–NCR | 1 | 1 | 1 | |
| | | 1–RT + CCNU | | | | 1 |
| | | Total (PPR group) | 2 | 1 | 1 | 1 |
| FAIL | 29 | 24 | | | | |
| | | 7–MVPP | | | | |
| | | 16–NCR | | 1 | 1 | |
| | | 1–RT + MVPP | 1 | | | |
| | | Total (Fail group) | 1 | 1 | 1 | |

RT = radiotherapy; NCR = non-cross-resistant regimen

**Table IV** Prognostic factors for remission induction

| *Prognostic Factor* | *Variable* | *Remission rate% by groups* | *Significance -univariate (P)* | *Significance multivariate (P)* |
|---|---|---|---|---|
| Albumin | <33g/l | 36 | <0.001 | 0.0002 |
| g/l | 33–39g/l | 61 | | |
| | >40g/l | 81 | | |
| Stage | IIIB | 75 | <0.001 | 0.004 |
| | IV | 50 | | |
| $\beta_2$ | <3>μg/ml | 64 | 0.002 | 0.02 |
| microglobulin | 3 + g/ml | 29 | | |
| μg/ml | <40:40 + | | 0.04 | NS |
| Age | years | | | |
| Sex | M:F | | NS | |
| ESR | <40:40 + | | NS | |
| Alkaline | Normal vs | | NS | |
| phosphatase | abnormal | | | |
| Lymphocyte | <0.75 × $10^9$/l | | NS | |
| count | >0.75 × $10^9$/l | | | |
| B symptoms | + :− | | NS | |
| Histology | | | NS | |

NS = not significant

On a reduced set of 60 patients with known $\beta_2$ microglobulin values, selected only for the fact that serum had been stored at presentation, using the logistic regression method, $\beta_2$ microglobulin was the only significant factor for achievement of CR ($P = 0.02$). The CR rate in patients with serum $\beta_2$ microglobulin levels in the normal range (0–3µg/ml) was 23/36 (64%), in contrast to 7/24 (29%) in individuals with elevated levels (>3µg/ml). This difference is highly significant on univariate ($P = 0.002$) and multivariate analysis ($P = 0.02$).

Age is significant by univariate analysis ($P = 0.04$), but is correlated with albumin and is not significant in the multivariate analysis. Lymphocyte count, histology, ESR, alkaline phosphatase and sex did not correlate with achievement of CR.

*First remission – CR* Fifty-five of 97 patients entering CR with six cycles of therapy remain in continuous remission, the median duration of first remission not having been reached (Figure 3). Twelve patients died in first remission. Recurrence has been documented in 30 between 0 and 13 years. Patients in GPR had a significantly higher 'recurrence' rate compared with patients who achieved CR ($P < 0.001$) (Figure 3), recurrence being documented in 15/18.

In these (remitters) patients, retrospective calculation of the cumulative dose administered [Appendix] demonstrated on univariate analysis, a significantly higher risk of recurrence, if there was a reduction of more than 15% of the planned cumulative dose over the first six cycles ($P = 0.009$).

However, the duration of remission was the same for patients who received maintenance chemotherapy after six cycles when compared with patients treated with six cycles only. There was no significant difference in remission duration of patients receiving two drug maintenance, compared to patients receiving four drug maintenance. (This has been previously reported as part of a multicentre Medical Research Council trial – MRC Working Party on Lymphomas, 1979.)

There is no significant difference in the duration of remission of patients who had additional irradiation, compared with patients who had chemotherapy alone.

Presentation age, gender, albumin, serum $\beta_2$ microglobulin, erythrocyte sedimentation rate (ESR), alkaline phosphatase, lymphocyte counts, B symptoms did not correlate with remission duration on univariate analyses.

**Figure 3** Duration of remission–CR v GPR.

**Figure 4** Survival of patients with advanced Hodgkin's disease A = uncensored; B = censored for second malignancy deaths only; C = censored for all non-Hodgkin's disease deaths.

*Recurrent disease*

Thirty patients had recurrent disease. Second CR was achieved in 17/30 and PR in two, with what was considered appropriate therapy (Table V). Overall, salvage therapy with non-cross-resistant regimens was more effective than MVPP type regimes at inducing second remission (Table VI)). The median duration of second remission is 4.3 years. Nine of those 17 who achieved second remission have had a second recurrence, third remission being reinduced in six, the median duration of third remission being 4.4 years.

Fifteen of the 18 patients in GPR have had recurrent progressive disease. Complete remission was induced in nine patients with appropriate salvage therapy (Table V) and GPR in a further three.

Response at first recurrence correlated with duration of first remission – remission (CR and GPR) was induced in 54% of patients whose first remission was less than 1 year, 63% when remission was between 1 and 2 years and 88% when remission greater than 2 years (Table VI). There was no correlation between age, gender, albumin level or extranodal disease at recurrence and achievement of second remission (or duration of second remission). Over a 15-year period, a significantly greater proportion of patients with recurrence within 2–3 years of second remission had duration of first remission of less that 12 months ($P = 0.01$). However, there was no overall correlation between duration of first remission and duration of second remission.

**Table V** Response to salvage therapy in patients with recurrent disease

| *Response to initial therapy* | *Salvage regimen* | *Number retreated* | *Response to salvage* CR | GPR | PPR |
|---|---|---|---|---|---|
| CR | MVPP Type | 12 | 5 | | |
| | NCR | 5 | 4 | 1 | |
| | RT | 8 | 7 | | |
| | Combined modality | 2 | 1 | | 1 |
| | No treatment | 3 | | | |
| | Total | 30 | 17 | 1 | 1 |
| GPR | MVPP type | 8 | 4 | 2 | 1 |
| | NCR | 5 | 4 | 1 | |
| | Surgery | 1 | 1 | | |
| | No treatment | 1 | | | |
| | Total | 15 | 9 | 3 | 1 |

NCR = non-cross-resistant therapy: ABVD = Sutcliffe *et al.* (1979) EVA = Richards *et al.* (1986).

**Table VI** Efficacy of salvage therapy related to time to recurrence

| *Time Interval* | *Total* | *MVPP* | *NCR* | *RT* | *Other* |
|---|---|---|---|---|---|
| <1 year | 7/13 | 3/8 | 1/1 | 2/2 | 1/2 |
| 1–2 years | 10/16 | 4/6 | 5/5 | 2/3 | 0/3 |
| >2 years | 14/16 | 6/7 | 5/5 | 3/3 | – |
| Total | 31/45 | 12/21 | 11/11 | 7/8 | 1/5 |

*Survival from initial presentation*

Eighty-two of 164 patients are still alive, the overall median survival being 13.7 years (Figure 4). Eighty-two patients have died; 66 patients had active HD at the time of death, 42 never having been in clinical remission (CR or GPR). The median survival of the non-remitters, excluding early deaths, is 0.4 years (0.2 years, including early deaths) (Figure 5a). Though the numbers are small, the survival of patients who achieve clinical remission with second line therapy or require more than six cycles of MVPP is significantly worse than patients who achieve clinical remission with initial chemotherapy ($P = 0.004$). The survival of patients in CR is the same as for patients in GPR, despite a significantly higher rate of recurrent disease in the latter (Figure 5b).

Fifty-five of the 97 patients entering CR are alive without ever having had a recurrence, 6/97 are alive having had one recurrence, four having had two recurrences and one having had three recurrences. Thirty-one of the 97 remitters are dead, 12 never having had a recurrence, 15 with recurrence once, three twice and one three times (Figure 6).

Sixteen patients died without clinical evidence of Hodgkin's disease at the time of death, 12 never having recurrent disease, four following recurrence and being in second (two) or third remission (two).

There was no significant difference in survival of patients treated with MVPP compared with patients treated with ChlVPP.

The stepwise linear regression method was used to detect any differences in survival correlating with age, sex, stage, histology, albumin, alkaline phospatase, absolute lymphocyte count or ESR. The only factors which significantly affect survival adversely on univariate and multivariate analysis are advanced age, histology (lymphocyte deplete) and low absolute lymphocyte count ($<0.75 \times 10^9/l$) (Table VII). Albumin failed to reach statistical significance (despite its importance for achievement of CR) though it correlates highly with lymphocyte deplete histology (mean albumin in patients with lymphocyte deplete histology is 31 g/l compared to 37 g/l in patients with other histology ($P = 0.001$, $t$-test) and low absolute lymphocyte count. Similarly, stage correlates with age (mean age in stage IIIB patients was 34 years, compared to 42 years for stage IV patients ($P = 0.001$, $t$-test)). Stage and albumin did not, therefore reach statistical significance in the multivariate analysis.

**Figure 5** **a**, Survival of remitters v non-remitters v patients who achieve remission with second line therapy. **b**, Survival of patients in CR v GPR v PR.

**Figure 6** Flow diagram of patterns of remission and recurrence.

**Table VIIa** Univariate analysis of prognostic factors for survival

| *Variable* | *Significance-P value* |
|---|---|
| Albumin | 0.002 |
| Age <45:46 + | <0.001 |
| Lymphocyte count <0.75:0.75 × 10⁹ l | 0.009 |
| Histology LD:rest | <0.001 |
| $\beta_2$microglobulin | 0.003 |
| Stage IIIB:IV | 0.01 |
| Maintenance chemotherapy | 0.1 |
| ChlVPP:MVPP | 0.4 |
| Chemotherapy:combined modality | 0.66 |

**Table VIIb** Multivariate analysis of prognostic factors for survival

| *Prognostic factor* | *All deaths* *RR* | *P* | *Non HD deaths censored* *RR* | *P* | *Non HD and second malignancy deaths censored* *RR* | *P* |
|---|---|---|---|---|---|---|
| Age (<45:>45years) | 3.1 | 0.0001 | 2.4 | 0.002 | 2.4 | 0.003 |
| Histology (LD *vs* rest) | 2.3 | 0.03 | 2.6 | 0.01 | 2.6 | 0.02 |
| Lymphocyte count (<0.75 vs>0.75 × $10^9$ l) | 1.9 | 0.03 | 1.9 | 0.04 | 2.1 | 0.03 |

Sex, stage, albumin, $\beta_2$microglobulin, alkaline phosphatase and ESR were not significant on multivariate analysis. RR = relative risk; LD = lymphocyte depletion histology.

On the reduced set of 60 patients with known $\beta_2$ microglobulin levels, age and lymphocyte count were again significant. Serum $\beta_2$ microglobulin was significant on univariate ($P = 0.003$, Figure 7), but not on multivariate analysis.

On the basis of these prognostic factors, patients can be divided into two distinct groups—a good prognostic group, in which none of the patients have any of the adverse factors (age less than 45 years, lymphocyte count more than $0.75 \times 10^9$/l and histology apart from lymphocyte depletion), and a poor prognostic group, where patients have one or more of the adverse factors. The difference between the two groups is statistically very significant ($P < 0.001$) (Figure 8).

*Survival following recurrence* The median survival from first recurrence is 4 years, being better when second remission was achieved than the rest. The median survival of patients who achieve second CR is 12 years. Advanced age correlated adversely with survival, (age greater than 40, $P = 0.04$; age greater than 45, $P < 0.009$; age greater than 50, $P = 0.009$). Extranodal disease at time of recurrence was a significant adverse prognostic factor on univariate but not on multivariate analysis. Gender, albumin level at recurrence or duration of first remission were not significant in correlating for better survival.

### *Causes of death*

Eighty-two of the original 164 patients have died, 15 before completion of planned chemotherapy and 51 with refractory or recurrent disease. Thus 66/82 (80%) of the deaths occurred in patients with evidence of active disease. There were 16 deaths in patients who had no evidence of active disease at the time of death, six of which were due to second malignancy.

The causes of death are shown in Table VIII. Details of deaths of patients who died with no evidence of HD are in Table IX.

*Second malignancies* Second malignancies have been recorded in 10 patients (Table X), six having died as a result. The commonest second malignancy has been non-Hodgkin's lymphoma; there has been only one documented case of acute myeloid leukaemia. Of the 67 patients in continuous first remission, seven have developed a second malignancy. Three of the seven had maintenance chemotherapy (24 patients had maintenance chemotherapy in total); three others had irradiation in addition to the chemotherapy (21 remitters received irradiation overall). There was one second malignancy in second remission and one in third; one patient developed acute leukaemia with concurrent progressive disease at second recurrence.

**Figure 7** Survival of patients with normal *v* elevated $\beta_2$microglobulin levels at presentation.

**Figure 8** Survival of patients with Hodgkin's disease, by prognostic group. Good prognostic group:age <45 years and lymphocyte count more than 0.75 x 109/1 and histology apart from lymphocyte depletion. Poor prognostic group:either age >45 years or lymphocyte count <0.75 x 109/1 or lymphocyte deplete histology.

### Discussion

This analysis provides further evidence of the enormouse long-term survival advantage conferred on patients with advanced Hodgkin's disease by the achievement of complete remission with MOPP-like cyclical combination chemotherapy, with more than 50% of them predicted to be alive 20 years later, and only one recurrence having been seen to date, after 10 years. In contrast, the survival of those for whom no remission was achieved was as bad as the natural history of the disease.

From this perspective, the first urgent priority is an improvement of the complete remission rate. This was lower (59%) with MVPP than has been recorded by others with

**Table VIII** Causes of death

| | |
|---|---|
| Hodgkin's disease present | 66(15)[a] |
| Second malignancy | 6(10)[b] |
| Cerebrovascular accident | 2 |
| Myocardial infarction | 3 |
| Infection | 3 |
| Coma | 1 |
| Unknown | |

[a]Early deaths; [b]total second malignancy

**Table IX** Causes of deaths in remission

| Patient | Age at death | Which remission | Cause | Time from Diagnosis (years) |
|---|---|---|---|---|
| 1DG | 51 | First | Coma | 8 months |
| 2AN | 62 | First | NHL CVA | 3 |
| 3DS | 61 | First | CVA | 7 |
| 4EA | 54 | First | Oat cell carcinoma of bronchus | 7.5 |
| 5GH | 76 | First | Bronchopneumonia old age (age 76 years) | 8 |
| 6PL | 47 | First | Adenocarcinoma of lung | 9 years |
| 7CG | 34 | Third | Myeloproliferative disorder | 11 |
| 8BAJ | 72 | First | Carcinoma of the prostate | 11 |
| 9BC | 42 | Third | Myocardial infarction | 13 |
| 10AG | 58 | First | Unknown | 13.8 |
| 11CRW | 80 | First | Bronchopneumonia | 14 |
| 12AE | 46 | First | Myocardial infarction | 14.3 |
| 13WPG | 65 | Second | ? second malignancy (Histology inconclusive) | 15.3 |
| 14JAB | 72 | First | Myocardial infarction | 17.5 |
| 15RDH | 60 | First | Carcinoma of the oesophagus | 18 |
| 16COL | 49 | Second | Non-Hodgkin's lymphoma | 20 |

**Table X** Second malignancies

| Second malignancy | Remission status | Treatment | Time from diagnosis (years) |
|---|---|---|---|
| NHL | Second CR | MVPP, COPP, CVPP | 13.8 |
| NHL | First CR | MVPP MRT | 12.6 |
| NHL | First CR | MVPP maintenance | 10.5 |
| Carcinoma of oesophagus | First CR | MVPP MRT | 10 |
| Squamous cell carcinoma of skin and carcinoma of the prostate | First CR | MVPP MRT | 9.4 |
| Myelodysplastic syndrome | Third CR | MVPP maintenance | 9.4 |
| Adenocarcinoma of lung | First CR | MVPP + TNI | 9 |
| Oat cell carcinoma of lung | First CR | MVPP | 6.4 |
| Acute myeloid leukaemia | Progressive disease at second recurrence | MVPP | 3.1 |
| NHL | First CR | MVPP maintenance | 2.8 |

NHL = non-Hodgkin's lymphoma; MRT = mantle radiotherapy; TNI = total nodal irradiation; COPP = cyclophosphamide, vincristine, procarbazine and prednisolone; CVPP = cyclophosphamide, vinblastine, procarbazine and prednisolone.

MOPP or similar regimens (Longo *et al.*, 1986; Selby *et al.*, 1990; Ranson *et al.*, 1991), although some of the difference may be accounted for by variations in the patient populations, particularly with respect to age and general debility (as reflected by hypoalbuminaemia), additional therapy or differences in the criteria for documenting complete remission. Regardless of this, however, it is clear that either persisting with the same treatment beyond six cycles, or changing it at that time to an alternative is not a fruitful approach since this only increased the complete remission rate to 62%, and more important, the survival of this 6% was markedly inferior to that of the initial 59%. Failure to demonstrate significant efficacy of the ABVD programme (Santro & Bonadonna, 1979) for patients with refractory disease at St Bartholomew's Hospital, in contrast to the experience in Milan (Santoro *et al.*, 1979; 1982), may well relate solely to the fact that it was 'left too late'. The equivalent efficacy of the ABVD and MOPP in inducing remission provided the rationale for testing non-cross-resistant and hybrid combinations (Santoro *et al.*, 1982; Klimo & Connors, 1985). Most, but not all, recent data have supported this approach for improving responsiveness, the most spectacular results coming from Vancouver, with a reported complete remission rate of 88% (Klimo & Connors, 1988) after hybrid chemotherapy and involved field irradiation where necessary. Failure to achieve complete remission in this way, of course, leaves little room for manoeuvre and raises the question of whether bone marrow ablative treatment is indicated for some patients in this setting. The present consensus seem to be that it is only likely to benefit those who are at least showing some evidence of response (Jagannath *et al.*, 1989; Carella *et al.*, 1988); dose escalation *per se* does not universally overcome intrinsic resistance. Clearly some indication from the presentation features of probability of conventional treatment failing would be most helpful. The data above indicate that advanced age and hypoalbuminaemia are the major adverse factors, neither of which would be likely to predispose well to intensification of therapy. It is more likely that the rate of response will be a useful indicator, but the logistics of repeated detailed re-evaluation during therapy are daunting.

Presentation serum $\beta_2$ microglobulin levels, available in a smaller set of patients, correlated with achievement of complete remission; it remained statistically significant on multivariate analysis though albumin and stage were not. Amlot and Adinolfi (1979) found correlation between presentation $\beta_2$ microglobulin levels and initial stage in patients with Hodgkin's disease. This trend was also confirmed by Hagberg *et al.* (1983) and Child *et al.* (1980). However, there was no correlation with achievement of remission or survival. Prognostic effect of $\beta_2$ microglobulin on response to therapy and also on subsequent survival has been documented in non-Hodgkin's lymphoma and myeloma, but not in Hodgkin's disease (Legros *et al.*, 1987; Han *et al.*, 1989; Hagberg *et al.*, 1983). The cause of elevated $\beta_2$ microglobulin levels is still speculative. It has been suggested that there is increased shedding due to decreased cell surface expression or due to increased cell turnover. Swan *et al.* (1989) suggested that patients with high $\beta_2$ microglobulin had absent cellular expression of Class I Major Histocompatibility Complex (MHC). Therefore the prognostic value of serum $\beta_2$ microglobulin may provide a crude and indirect assessment of the importance of tumour MHC expression.

The freedom from recurrence pattern for those entering complete remission is very similar to that reported in the literature, with most recurrences occurring during the first 3 years, and approximately two-thirds still free of disease 10-15 years from presentation. Limited support for the importance of dose-intensity comes from the finding that reduction of more than 15% of the planned cumulative dose over the first six cycles correlated with a high risk of recurrence. This interpretation is of course complicated by the inevitable problem of retrospective analysis. Further, the fact that the MVPP programme is given every 6 weeks compared with 4 weeks for MOPP, and yields identical results in terms of freedom from recurrence suggests that there may well be a threshold above which the dose intensity effect becomes irrelevant. Whether the somewhat lower remission rate in this study compared with 4-weekly MOPP may reflect the effect of lower dose intensity is a matter of speculation. Preliminary results from a Cancer and Leukaemia Group B (CALGB) study suggest an advantage for ABVD in both inducing and maintaining remission (Canellos *et al.*, 1988; Anderson *et al.*, 1990) are interesting and await confirmation. Significant improvement in the complete remission, freedom from progression, relapse-free survival and overall survival with alternating MOPP ABVD over MOPP alone has been reported from Milan, though at a cost of increased treatment related toxicity (Bonadonna *et al.*, 1986).

Survival from recurrence was surprisingly good, and once again correlated most closely with the response to salvage therapy, and was characterised for most by repeated recurrences with a diminishing likelihood of response. The response rate was better following longer first remissions. The absence of an absolute correlation between duration of first remission and duration of second remission is surprising, although the majority of patients with second recurrence within 2–3 years of apparently successful re-induction therapy had had initial remission lasting less than 1 year. This weak correlation, the fact that the median duration of second remission was 4 years coupled with a median survival of 4 years from recurrence and 12 years from second remission makes selection of the most appropriate management at recurrence difficult, particularly as advancing age (above 40 years) was the only adverse feature identified. It is not yet clear whether survival following recurrence after non-cross-resistant alternating or hybrid chemotherapy will follow the same pattern as following MOPP or MVPP. Further newer combinations have been shown to be promising (Richards *et al.*, 1986).

The published experience with bone marrow ablative therapy is certainly encouraging, either if used at recurrence or as consolidation of remission (Carella *et al.*, 1988; Gribben *et al.*, 1989; Jagannath *et al.*, 1989; Jones *et al.*, 1990). However, much more data will be required to demonstrate its general applicability and efficacy. It is less, rather than more, likely to be relevant to most of the patients who have a recurrence when over 45 years of age, and this is the group in greatest need of help. It will clearly take many years to prove a survival advantage for such treatment at first recurrence or in second remission: the only valid endpoints of present studies in the near future will have to be freedom from recurrence and toxicity, with the hope that if the former is substantially improved, it will convert into longer survival. Whether it will be possible to answer the question without randomised trials is arguable. Minimisation of the short-term toxicity of treatment, possibly with the use of haemopoietic growth factors would clearly be most helpful and widen the range of people in whom therapy could be tested. While it can be postulated that the successful induction of a second remission is not an argument for very intensive consolidation, it might well be considered the treatment of choice following subsequent recurrences. The necessity of identifying the most appropriate patients to receive experimental therapy is obvious.

The pattern of survival for the whole population was obviously dominated by failure of the treatment, with 80% of the deaths so far having occurred in people with active disease. It has previously been reported that almost all of the men and at least half of the women who survive are sterile (Sherins & DeVita 1973; Chapman *et al.*, 1979; 1981; Waxman *et al.*, 1982) and with longer follow-up it is clear that the incidence of second malignancy continues to increase (Tester *et al.*, 1984; Dorreen *et al.*, 1986; Coleman, 1986; Tucker *et al.*, 1988; Kaldor *et al.*, 1990; Somers *et al.*, 1990). Second malignancies accounted for 10% of the deaths in remitters, being the single most important cause of mortality after HD. The risk of second malignancy seems to be increased in patients who receive additional therapy—6 7 remitters who develop second malignancy had had extra chemotherapy as maintenance or additional irradiation. Three other patients who developed second malignancy following recurrence had been retreated with combination chemotherapy. The increased risk of secondary malignancy with increasing therapy is well recognised, particularly following irradiation (Coleman, 1986; Somers *et al.*, 1990). Data on more than 12,411 patients treated for HD in different centres were pooled and analysed in 1989 (Somers *et al.*, 1990). The cumulative incidence of second malignancy over a 20-year follow-up period approaches 18.6%, with 'solid tumours' accounting for the majority of late malignancies. In this group of patients treated with MVPP with a minimum follow-up of 6 years there has only been one instance of frank acute myeloid leukaemia which occurred at the same time as progressive Hodgkin's disease at second recurrence, and one myelodysplastic syndrome in third remission. In the NCI series (Longo *et al.*, 1986), there were 13 cases of acute leukaemia, from a total cohort of 198 patients, 12 of which occurred in patients who had received both MOPP and irradiation. In contrast to these results, Kaldor *et al.* (1990), in a case controlled study of 163 cases of leukaemia following therapy for Hodgkin's disease, found no increased risk in patients receiving additional irradiation. In the same study, treatment with more than six cycles significantly increased the risk of secondary acute leukaemia. The Stanford data suggest that the risk of acute leukaemia reaches a plateau at 10 years, at 3.3%, though the risk of secondary non-Hodgkin's lymphoma continues to increase. The cumulative actuarial risk of all second cancers from the Stanford series is 17.6% at 15 years (Tucker *et al.*, 1988). The cumulative risk of acute leukaemia in this present series is thus lower than that reported with a number of series using MOPP. Whether this is a consequence of the 'lower' intensity with which MVPP is administered, being 6-weekly instead of 4-weekly, needs to be determined.

The long-term toxicity of the newer treatments, either without alkylating agents, or including them in lower doses in the form of hybrid or alternating programmes, may well be much less than that of MOPP alone. Much will have been gained even if this is the only achievement of modifying the initial therapy. Reducing its duration was certainly a benefit. Neither the failure of MOPP like therapy to eliminate HD from some patients nor its late toxicity, even fatal for others, should be allowed to detract from the significant benefit it has brought to the majority of those who received it. The challenge is to look forward and do better.

**Appendix**

*Dose reduction*

Percentage of planned dose administered

$$= \frac{\frac{T}{P}\text{ Mustine} + \frac{T}{P}\text{ Vinblastine} + \frac{T}{P}\text{ Procarbazine} + \frac{T}{P}\text{ Prednisolone}}{4} \times 100$$

T = total dose administered. P = planned dose.

**References**

AMLOT, P. & ADINOLFI, M. (1979). $\beta_2$ microglobulin and its prognostic value in lymphomas. *Eur. J. Cancer.*, **15**, 791–796.

ANDERSON, J.R., CANELLOS, G.P., PROPERT, K.J. & others (1990). MOPP vs ABVD vs MOPP alternating with ABVD as treatment for advanced Hodgkin's disease: results at a median follow up of 4 years. Fourth International Conference on Malignant Lymphoma. June 6-9, p.26.

ARMITAGE, P. (1971) *Statistical Methods in Medical Research*. Halstead: London.

BONADONNA, G., VALAGUSSA, P. & SANTORO, A. (1986). Alternating non cross resistant combination chemotherapy or MOPP in stage IV Hodgkin's disease. *Ann. Intern. Med.*, **104**, 739–746.

CANELLOS, G.P., PROPERT, K., COOPER, R. & others (1988). MOPP vs ABVD vs MOPP alternating with ABVD in advanced Hodgkin's disease: a prospective randomized CALGB trial. *Proc. Annu. Meet. Am. Soc. Clin. Oncol.*, **7**, A888.

CARBONE, P.P., KAPLAN, H.S., MUSSHOFF, K. & others (1971). Report of the committee on Hodgkin's disease staging classification. *Cancer Res.*, **31**, 1860.

CARELLA, A.M., CONGIU, A.M., GAOZZA, E. & others (1988). High dose chemotherapy with autologous bone marrow transplantation in 50 advanced resistant Hodgkin's disease patients: an Italian Study Group report. *J. Clin. Oncol.*, **6**, 1411–1416.

CHAPMAN, R.M., SUTCLIFFE, S.B. & MALPAS, J.S. (1979). Cytotoxic induced ovarian failure in Hodgkin's disease. *J. Am. Med. Ass.*, **242**, 1882–1884.

CHAPMAN, R.M., SUTCLIFFE, S.B. & MALPAS, J.S. (1981). Male gonadal dysfunction in Hodgkin's disease. A prospective study. *J. Am. Med. Ass.*, **245**, 1323–1328.

CHILD, J., SPATI, B., ILLINGWORTH, S. & others. (1980). Serum $\beta_2$ Microglobulin and C-reactive protein in the monitoring of lymphomas. *Cancer*, **45**(2), 318-326.

COLEMAN, C.N. (1986). Secondary malignancy after treatment of Hodgkin's disease: an evolving picture. *J. Clin. Oncol.*, **4**, 821–824.

COX, D.R. (1972). Regression models and life tables. *J. Roy. Stat. Soc.*, **34**, 187–220.

DEVITA, V.T., SERPICK, A.A. & CARBONE, P.P. (1970). Combination chemotherapy in the treatment of Advanced Hodgkin's disease. *Ann. Intern. Med.*, **73**, (6), 881–895.

DORREEN, M.S., GREGORY, W.M., WRIGLEY, P.F.M., STANSFELD, A.G. & LISTER, T.A. (1986). Second primary malignant neoplasms in patients treated for Hodgkin's disease at St. Bartholomew's Hospital. *Hematol. Oncol.*, **4**, 149–161.

FISHER, R.I., DEVITA, V.T., HUBBARD, S.P. & others (1979). Prolonged disease free survival in Hodgkin's disease with MOPP reinduction after first relapse. *Ann. Intern. Med.*, **90**, 761-763.

GRIBBEN, J.G., LINCH, D.C., SINGER, C.R.J. & others (1989). Successful treatment of refractory Hodgkin's disease by high dose combination chemotherapy and autologous bone marrow transplantation. *Blood*, **73**, (1), 340-344.

HAGBERG, H., KILLANDER, A. & SIMONSSON, B. (1983). $\beta_2$ Microglobulin in malignant lymphoma. *Cancer*, **51**,(12), 2220-2225.

HAN, T., BHARGAVA, A., HENDERSON, E. & others (1989). Prognostic significance of $\beta_2$ microglobulin in chronic lymphocytic leukaemia and non-Hodgkin's lymphoma. *Proc. ASCO*, **8**, 1056.

HANCOCK, B.W. (1986). Randomised study of MOPP against LOPP in advanced Hodgkin's disease. *Radiother. Oncol.*, **7**, 215-221.

HORNING, S.J., HOPPE, R.T., KAPLAN, H.J. & others (1981). Female reproductive potential after treatment for Hodgkin's disease. *N. Engl. J. Med.*, **304**, 1377.

JAGANNATH, S., ARMITAGE, J.O., DICKE, K.A. & others (1989). Prognostic factors for response and survival after high dose cyclophosphamide, carmustine and etoposide with autologous bone marrow transplantation for relapsed Hodgkin's disease. *J. Clin. Oncol.*, **7**, 179-185.

JONES, R.J., PIANTADOSI, S., MANN, R.B. & others (1990). High dose cytotoxic therapy and bone marrow transplantation for relapsed Hodgkin's disease. *J. Clin. Oncol.* **8**, (3): 527-537.

KALDOR, J.M., DAY, N.E., CLARKE, E.A. & others (1990). Leukaemia following Hodgkin's disease. *N. Engl. J. Med.*, **322**, 7-13.

KAPLAN, E.L. & MEIER, P. (1958). Non parametric observations from incomplete observations. *Am. Stat. Assoc. J.*, **53**, 457-80.

KLIMO, P. & CONNORS, J.M. (1985). MOPP/ABV Hybrid program: combination chemotherapy based on early introduction of seven effective drugs for advanced Hodgkin's disease. *J. Clin. Oncol.*, **3**, 1174-1182.

KLIMO, P. & CONNORS, J.M. (1988). An update on the Vancouver experience in the management of advanced Hodgkin's disease treated with the MOPP/ABV Hybrid program. *Sem. Hemato.*, **25**, (2) (Suppl. 2), 34-40.

LEGROS, M., FERRIER, J., BIGNON, Y. & others (1987). $\beta_2$ microglobulin: a good prognosis indicator in non Hodgkin's lymphoma. *Proc. ASCO*, **6**, 748.

LISTER, T.A., CROWTHER, D., SUTCLIFFE, S.B. & others (1989). Report of a committee convened to discuss the evaluation and staging of patients with Hodgkin's disease; Cotswolds meeting. *J. Clin. Oncol.*, **7**, 1630-1636.

LONGO, D.L., YOUNG, R.C., WESLEY, M. & others (1986). Twenty years of MOPP therapy for Hodgkin's disease. *J. Clin. Oncol.*, **4**, 1295-1306.

McKENDRICK, J.J., MEAD, G.M., SWEETENHAN, J. & others (1989). ChlVPP chemotherapy in advanced Hodgkin's disease. *Eur. J. Cancer Clin. Oncol.*, **25**, (3), 557-561.

MEDICAL RESEARCH COUNCIL (1979). Working Party on Lymphomas: Randomised Trial of Two-Drug and Four-Drug Maintenance Chemotherapy in Advanced or Recurrent Hodgkin's Disease. *Br. Med. J.*, **1**, 1105-1108.

NICHOLSON, W.M., BEARD, M.E.J., CROWTHER, D. & others (1970). Combination chemotherapy in generalized Hodgkin's disease. *Br. Med. J.*, **3**, 7-10.

PETO, R., PIKE, M.C., ARMITAGE, P. & others (1977). Design and analysis of randomized clinical trials requiring prolonged observation of each patient. *Br. J. Cancer.*, 351-77.

RANSON, M.R., RADFORD, J.A., SWINDELL, R. & others (1991). An analysis of prognostic factors in stage III and IV Hodgkin's disease treated at a single centre with MVPP. *Ann. Oncol.*, **2**, 423-429.

RICHARDS, M.A., WAXMAN, J.H. & MAN, T. (1986). EVA treatment for recurrent or unresponsive Hodgkin's disease. *Cancer Chemother. Pharmacol.*, **18**, 51-53.

SANTORO, A. & BONADONNA, G. (1979). Prolonged disease free survival in MOPP resistant Hodgkin's disease after treatment with adriamycin, bleomycin, vinblastine and dacarbazine (ABVD). *Cancer Chemother. Pharmacol.*, **2**, 101-5.

SANTORO, A., BONFANTE, V. & BONADONNA, G. (1982). Salvage chemotherapy with ABVD in MOPP-resistant Hodgkin's disease. *Ann. Intern. Med.*, **96**, 139-143.

SELBY, P., PATEL, P., MILAN, S. & others (1990). ChlVPP combination chemotherapy for Hodgkin's disease: long term results. *Br. J. Cancer*, **62**, 279-285.

SHERINS R.J. & DEVUTA, V.T. (1973). Effect of drug treatment for lymphoma on male reproductive capacity: studies of men in remission after therapy. *Ann. Intern. Med.*, **79**, 216-220.

SOMERS, R., HENRY-AMAR, M., MEERWALDT, J.K. & CARDE, P. (eds) (1990). *Treatment Strategy in Hodgkin's Disease*. Colloque INSERM/John Libbey Eurotext Ltd., vol 196.

SUTCLIFFE, S.B., WRIGEY, P.F.M., STANSFELD, A.G. & MALPAS, J.S. (1979). ABVD therapy for advanced Hodgkin's disease resistant to MVPP. *Cancer Chemother. Pharmacol.*, **2**, 209-213.

SUTCLIFFE, S.B., WRIGLEY, P.F.M., PETO, J. & others (1978). MVPP chemotherapy regimen for advanced Hodgkin's disease. *Br. Med. J.*, **1**, 679-683.

SWAN, F., VELASQUEZ, W., TUCKER, S. & others (1979). A new serologic staging system for large-cell lymphomas based on initial $\beta_2$ microglobulin and lactate dehydrogenase levels. *J. Clin. Oncol.*, **7**, (10), 1518-1527.

TESTER, W.J., KINSELLA, T.J., WALLER, B. & others (1984). Second malignant neoplasms complicating Hodgkin's disease: the National Cancer Institute experience. *J. Clin. Oncol.*, **2**, 762-769.

TUCKER, M.A., COLEMAN, C.N., COX, R.S. & others (1988). Risk of second cancers after treatment for Hodgkin's disease. *N. Engl. J. Med.*, **318**, 76-81.

WAXMAN, J., TERRY, Y.A., WRIGLEY, P.F.M. & others (1982). Gonadal function in Hodgkin's disease: long term follow up of chemotherapy. *Br. Med. J.*, **285**, 1612-1613.